# Urinary Concentrations of Parabens and Serum Hormone Levels, Semen Quality Parameters, and Sperm DNA Damage

**DOI:** 10.1289/ehp.1002238

**Published:** 2010-09-28

**Authors:** John D. Meeker, Tiffany Yang, Xiaoyun Ye, Antonia M. Calafat, Russ Hauser

**Affiliations:** 1 Department of Environmental Health Sciences, University of Michigan School of Public Health, Ann Arbor, Michigan, USA; 2 Centers for Disease and Control and Prevention, Atlanta, Georgia, USA; 3 Department of Environmental Health, Harvard School of Public Health, Boston, Massachusetts, USA; 4 Vincent Memorial Obstetrics and Gynecology Service, Massachusetts General Hospital, Boston, Massachusetts, USA

**Keywords:** biomarker, endocrine, epidemiology, exposure, fertility, reproduction, thyroid

## Abstract

**Background:**

Parabens are commonly used as antimicrobial preservatives in cosmetics, pharmaceuticals, and food and beverage processing. Widespread human exposure to parabens has been recently documented, and some parabens have demonstrated adverse effects on male reproduction in animal studies. However, human epidemiologic studies are lacking.

**Objective:**

We investigated relationships between urinary concentrations of parabens and markers of male reproductive health in an ongoing reproductive epidemiology study.

**Methods:**

Urine samples collected from male partners attending an infertility clinic were analyzed for methyl paraben (MP), propyl paraben (PP), butyl paraben (BP), and bisphenol A (BPA). Associations with serum hormone levels (*n* = 167), semen quality parameters (*n* = 190), and sperm DNA damage measures (*n* = 132) were assessed using multivariable linear regression.

**Results:**

Detection rates in urine were 100% for MP, 92% for PP, and 32% for BP. We observed no statistically significant associations between MP or PP and the outcome measures. Categories of urinary BP concentration were not associated with hormone levels or conventional semen quality parameters, but they were positively associated with sperm DNA damage (*p* for trend = 0.03). When urinary BPA quartiles were added to the model, BP and BPA were both positively associated with sperm DNA damage (*p* for trend = 0.03). Assessment of paraben concentrations measured on repeated urine samples from a subset of the men (*n* = 78) revealed substantial temporal variability.

**Conclusions:**

We found no evidence for a relationship between urinary parabens and hormone levels or semen quality, although intraindividual variability in exposure and a modest sample size could have limited our ability to detect subtle relationships. Our observation of a relationship between BP and sperm DNA damage warrants further investigation.

Parabens are esters of 4-hydroxybenzoic acid that are commonly used as antimicrobial preservatives in cosmetics, pharmaceuticals, and food or beverage processing (Andersen 2008). It is likely that repeated contact with products or foods containing parabens leads to widespread human exposure through ingestion, inhalation, or dermal contact. Methyl paraben (MP), ethyl paraben (EP), propyl paraben (PP), and butyl paraben (BP) have recently been detected in a high proportion of urine samples collected as part of a national U.S. survey of exposure to environmental chemicals (Calafat et al. 2010).

Parabens are thought to possess low toxicity (Golden et al. 2005; Soni et al. 2005). However, some parabens may be estrogenic *in vitro* (Routledge et al. 1998), although with activity levels several orders of magnitude lower than that of estrogen. Recent experimental studies have reported that certain parabens may also act as antiandrogens (Chen et al. 2007; Darbre and Harvey 2008; Satoh et al. 2005), and there is limited evidence that parabens may affect thyroid function (Rousset 1981; Taxvig et al. 2008; Vo et al. 2010). Several animal studies have explored the impacts of parabens on male reproductive effects, with some reporting that exposure to BP and PP, but not MP, adversely affects spermatogenesis and endocrine function in rats or mice (Kang et al. 2002; Oishi 2001, 2002a; 2002b; 2004). Conversely, in another recent study, Hoberman et al. (2008) found no association between MP or BP and reproductive markers in rats. Based on these limited animal studies, in addition to the observation that paraben estrogenicity depends on the length of the alkyl side chain (Darbre and Harvey 2008), the reproductive toxicity potential of parabens for which widespread human exposure has been documented is thought to be BP > PP > EP > MP.

To our knowledge, no human studies have investigated the association between paraben exposure and measures of male reproduction. In the present study we assessed relationships between urinary concentrations of several parabens and a range of male reproductive health markers in an ongoing study of environmental determinants of reproductive health. We also considered the potential for interactions between parabens and another estrogenic xenobiotic, bisphenol A (BPA), for which we recently reported associations with reproductive measures in this population (Meeker et al. 2010a, 2010b).

## Materials and Methods

Participants were male partners in subfertile couples seeking treatment between 2000 and 2004 from the Vincent Memorial Obstetrics and Gynecology Service. After the study procedures were explained and all questions answered, subjects signed an informed consent. Men between 18 and 55 years of age without postvasectomy status who presented to the Andrology Laboratory were eligible to participate. Of those approached, approximately 65% consented. Most men who declined to participate in the study cited lack of time on the day of their clinic visit as the reason for not participating. The study was approved by the human studies institutional review boards of MGH, Harvard School of Public Health, the Centers for Disease Control and Prevention (CDC), and the University of Michigan.

On the day of each subject’s clinic visit, a single spot urine sample was collected into a sterile polypropylene cup. Because the temporal reliability of parabens in urine is unknown, second and third urine samples were collected from a subset of men at subsequent clinic visits. These samples were generally collected between 1 week and 2 months after the first sample. After measuring specific gravity (SG) using a handheld refractometer (National Instrument Co. Inc., Baltimore, MD), each urine sample was divided in aliquots and frozen at −80°C. Samples were shipped on dry ice overnight to the CDC, where concentrations of total (free + conjugated) MP, PP, BP, and BPA were measured using a modification of the approach described by Ye et al. (2006). EP was not measured because of low detection rates among the U.S. population (Calafat et al. 2010). The analytical method and results for BPA have been previously reported (Meeker et al. 2010a, 2010b). The conjugated species of BPA and parabens were first hydrolyzed in 100 μL of urine using β-glucuronidase/sulfatase (*Helix pomatia*, H1; Sigma Aldrich, St. Louis, MO). The target compounds were preconcentrated by online solid-phase extraction, separated from other urine components by reversed-phase high-performance liquid chromatography, and detected by atmospheric pressure chemical ionization/isotope dilution/tandem mass spectrometry with peak focusing (Ye et al. 2006). The limits of detection (LODs) were 1.0 μg/L for MP and 0.2 μg/L for PP and BP. Low-concentration (2.2–9.0 μg/L) and high-concentration (10.5–53.8 μg/L) quality control materials (QCLs and QCHs, respectively) were prepared with pooled human urine that was analyzed with standards, reagent blanks, and study samples. The precision of measurements, expressed as the relative SD of 55–66 measures, depending on the analyte, was 5.8–12.1% for QCLs and 4.4–5.6% for QCHs. Paraben concentrations were corrected for SG using the formula


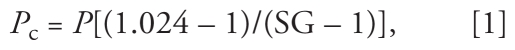


where *P*_c_ is the SG-adjusted paraben concentration (nanograms per milliliter), *P* is the observed paraben concentration, and SG is the specific gravity of the urine sample.

One nonfasting blood sample was drawn between 0900 and 1600 hours on the same day and time that the first urine sample was collected. Blood samples were centrifuged, and the resulting serum was stored at −80°C until hormone analysis at the MGH Reproductive Endocrinology Laboratory. Serum testosterone, estradiol (E_2_), sex-hormone– binding globulin (SHBG), inhibin B, follicle-stimulating hormone (FSH), luteinizing hormone (LH), prolactin, free thyroxine (T_4_), total triiodothyronine (T_3_), and thyroid- stimulating hormone (TSH) were measured using sensitive immunoassay methods as described previously (Meeker et al. 2007). The free androgen index (FAI) was calculated as the molar ratio of total testosterone to SHBG. Free testosterone was also estimated using published methods (Vermeulen et al. 1999). The testosterone:LH ratio, a measure of Leydig cell function, was calculated by dividing testosterone (nanomoles per liter) by LH (international units per liter). The FSH:inhibin B and E_2_:testosterone ratios were also calculated as measures of Sertoli cell function and aromatase activity, respectively.

Onsite at MGH, semen was collected from each subject into a sterile plastic specimen cup after a recommended abstinence period of 48 hr. After liquefaction at 37°C for 20–60 min, semen quality parameters and motion characteristics were measured at the clinic.

Semen samples were analyzed for sperm concentration, motility, and motion parameters using a computer-aided semen analyzer (CASA; version 10HTM-IVOS; Hamilton-Thorne Research, Beverly, MA) as previously described (Meeker et al. 2004a, 2008). Total sperm count (10^6^ per ejaculate) was calculated by multiplying sperm concentration (10^6^/mL) by semen sample volume (milliliters). Motile sperm was defined as World Health Organization (WHO) grade “a” sperm (rapidly progressive with a velocity ≥ 25 μm/sec at 37°C) plus “b” grade sperm (slow/sluggish progressive with a velocity of ≥ 5 μm/sec but < 25 μm/sec) (WHO 1999). Of seven CASA motion variables measured, we included only three in our analysis [straight-line velocity (VSL), curvilinear velocity (VCL), and linearity (VSL/VCL × 100)] because of a high degree of dependence between several of the measures (Duty et al. 2004; Meeker et al. 2004a). Sperm morphology was assessed on two slides per specimen (with a minimum of 200 cells assessed per slide) with a Nikon microscope using an oil-immersion 100× objective (Nikon Company, Tokyo, Japan). We used strict Kruger scoring criteria to classify men as having normal or below normal morphology (Kruger et al. 1988).

The remaining unprocessed semen was frozen in 0.25 mL cryogenic straws (Cryo Bio System, IMV Technologies, San Diego, CA) by immersing the straws directly into liquid nitrogen (−196°C). Previous work in our laboratory showed that this freezing method produced comet assay results that were highly correlated with results from fresh, unfrozen samples (Duty et al. 2002). Semen samples were later analyzed in batches, where straws were thawed by gently shaking in a 37°C water bath for 10 sec, and the semen was immediately processed for the comet assay. To assess sperm DNA damage, we followed a comet assay procedure that has been previously described (Duty et al. 2003; Singh and Stephens 1998). After lysis, electrophoresis, and staining, we measured comet extent, tail distributed moment (TDM), and percent DNA located in the tail (Tail%) for 100 sperm in each semen sample using a fluorescence microscope and VisComet software (Impuls Computergestutzte Bildanalyse GmbH, Gilching, Germany). Comet extent is a measure of total comet length from the beginning of the head to the last visible pixel in the tail. Tail% is a measurement of the proportion of total DNA that is present in the comet tail (i.e., fragmented DNA that has migrated away from the comet head). TDM is an integrated value that takes into account both the distance and intensity of comet fragments:


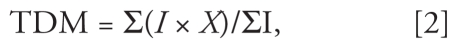


where ∑*I* is the sum of all intensity values that belong to the head, body, or tail and *X* is the x-position of the intensity value.

We performed data analysis using SAS (version 9.1; SAS Institute Inc., Cary, NC). In preliminary data analysis, paraben concentrations and outcome measures were compared by demographic categories and other potentially important covariates using appropriate parametric or nonparametric tests to investigate the potential for confounding. Multivariable linear regression was used to explore relationships between urinary paraben concentrations and hormone levels, semen quality parameters, and sperm DNA damage measures. Serum concentrations of inhibin B, testosterone, free testosterone, and E_2_, along with semen volume, sperm motility, morphology, and all sperm motion and sperm DNA damage measures, closely approximated normality and were used in statistical models untransformed. The distribution of sperm count, sperm concentration, FSH, LH, SHBG, prolactin, TSH, and all calculated hormone ratios were positively skewed and transformed by the natural logarithm (ln) for statistical analyses. Urinary MP and PP concentrations were also ln transformed. For PP, MP, and BPA concentrations < LOD, we used an imputed value of LOD/2. Because of the high proportion of samples with BP values < LOD, a three-level ordinal variable was formed: all samples with concentrations < LOD were assigned to the lowest group, and two equally sized groups were formed among the samples with detectable concentrations to form the median- and high-exposure groups. Tests for trend were conducted for ordinal BP categories in regression models using integer values (0, 1, 2).

Inclusion of covariates in the multivariable models was based on statistical and biologic considerations (Kleinbaum et al. 1998). We included SG as a continuous variable in all models to adjust for urinary dilution. Age and body mass index (BMI) were also modeled as continuous variables, whereas abstinence period was treated as an ordinal categorical variable. Race (white vs. other), smoking status (current smoker vs. former or never smoker), and timing of the clinic visit (i.e., time of collection of urine/blood/semen samples) by season (winter vs. spring, summer, or fall) and by time of day (0900–1259 hours vs. 1300–1600 hours) were considered for inclusion in the models as dichotomous variables. Covariates with a *p*-value < 0.2 in their relationship with one or more parabens or at least one outcome measure in the preliminary bivariate analyses were included in a “full” model. Covariates with a *p*-value > 0.15 in full models for all measures within the three sets of outcomes (hormone levels, semen quality, sperm DNA damage) were removed from the final models. Models for all outcome measures within each of the three sets of outcomes were adjusted for the same covariates to maintain consistency. For the dependent variables of interest (urinary paraben concentrations), we considered *p* < 0.05 statistically significant. Because of the exploratory nature of the analysis, *p*-values < 0.1 were considered statistically suggestive.

Because repeated urine samples were available for a subset of the men, two sets of models were constructed: *a*) using only urinary paraben concentrations from a single urine sample collected on the same day as the serum sample; and *b*) using the geometric mean urinary paraben concentration for each participant, where between one and three values were used to calculate each individual’s geometric mean (i.e., the geometric mean for men with only one value was equal to that single value). In further sensitivity analyses, the multivariable models were rerun after excluding men with highly concentrated or highly dilute urine samples (SG > 1.03 or < 1.01) (Teass et al. 1998), and when using SG-corrected paraben concentrations rather than uncorrected urinary paraben concentrations but including SG as a covariate. Finally, to assess temporal variability in urinary paraben concentrations, we calculated Spearman correlations for paraben concentrations in the first and second urine samples among men with two urine samples. Using paraben data from all men, we also calculated the intraclass correlation coefficient (ICC), which is the ratio of between-subject variability to total variability (total variability = between-subject variability + within-subject variability), using SAS PROC MIXED.

## Results

Demographic and outcome measure distribution data on the men who participated in the study have been described elsewhere (Meeker et al. 2010a, 2010b). Briefly, most men were white (84%) and were nonsmokers at the time of the clinic visit (90%). The mean (± SD) age and BMI were 36.7 ± 5.4 years and 27.4 ± 4.5, respectively. Median (25th, 75th percentile) values for sperm concentration, motility, and morphology were 100 million (24 million, 145 million) sperm/mL, 46% (26%, 66%) motile sperm, and 7% (3%, 10%) normal sperm, respectively.

Detection rates and distributions of uncorrected and SG-corrected urinary paraben concentrations from urine samples collected from 194 men on the same day as a serum sample (hormone levels) and/or semen sample (semen quality, sperm DNA damage) are presented in Table 1. Information on SG was missing for four urine samples. We detected MP in all samples and at the highest concentrations (median = 27.4 μg/L), followed by PP (median = 3.5 μg/L). BP was positively associated with age (Spearman *r* = 0.16; *p* = 0.03), but MP and PP were not. All three parabens were inversely associated with BMI (Spearman *r*, between −0.15 and −0.17; *p*-values < 0.05). Paraben concentrations were not associated with smoking status, time of day or season of urine sample collection, or duration of abstinence before semen sample collection. PP concentrations (*p* < 0.05), but not MP or BP concentrations (*p* > 0.05), were higher in nonwhite men than in white men. MP and PP concentrations were strongly correlated with one another (*r* = 0.82; *p* < 0.0001), and both were only weakly to moderately correlated with BP (both *r* = 0.39; *p* < 0.0001). Spearman correlation coefficients with urinary BPA were 0.29 for MP (*p* < 0.0001), 0.18 for PP (*p* = 0.01), and 0.11 for BP (*p* = 0.13).

In addition to the 194 urine samples collected at the same visit as serum/semen sample collection, a second urine sample was later collected from 78 of the men, and a third urine sample was collected from 4 men. The amount of time between consecutive urine samples ranged from 3 to 75 days, with a median (25th, 75th percentile) of 29 (27, 34) days. Among the 78 men who provided two urine samples, paraben concentrations in the first and second urine samples were weakly correlated for MP (Spearman *r* = 0.36; *p* = 0.003) and PP (*r* = 0.25; *p* = 0.03), and moderately correlated for BP (*r* = 0.46; *p* < 0.0001). However, these values increased when using SG-corrected concentrations (*r* = 0.46, 0.31, and 0.58, respectively). When considering all 272 urine samples with paraben concentrations measured, the ICC was 0.26 (SG-corrected, 0.35) for MP and 0.18 (SG-corrected, 0.26) for PP. We did not calculate ICC for BP because of the high proportion of nondetectable concentrations. For comparison, the ICC for BPA in these samples was 0.08 (SG-corrected, 0.13).

Urinary paraben, covariate, and outcome data were available from 167 men for hormone levels, 190 men for semen quality parameters, and 132 men for sperm DNA damage measures. In exploratory bivariate analyses, we found no statistically significant correlations between uncorrected or SG-corrected concentrations of MP or PP and hormone levels, semen quality parameters, or sperm DNA damage (data not shown). We also found no associations in multivariable linear regression models adjusted for age, BMI, smoking, time of day, and duration of abstinence (Tables 2 and 3), although we found a suggestive inverse relationship between MP and TSH (Table 2), a suggestive positive association between MP and Tail% (Table 3), and a suggestive inverse relationship between PP and TDM (Table 3). Results were similar when we also considered repeated urinary paraben concentrations measured in samples collected weeks or months after the initial urine sample, although the relationships between MP and TSH and Tail%, and between PP and TDM, were somewhat weakened (i.e., regression coefficients were closer to zero; data not shown). Our findings were also consistent when using SG-corrected paraben concentrations in the models, as well as when excluding concentrated or diluted urine samples with SG > 1.03 or < 1.01 (data not shown).

**Figure 1:**
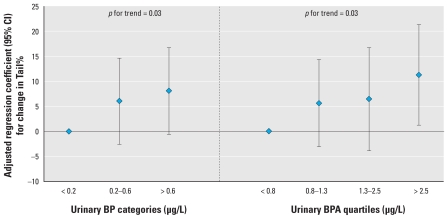
Adjusted linear regression coefficients [95% confidence interval (CI)] for change in Tail% associated with categories of urinary BP and BPA included in the same model, adjusted for SG, age, BMI, abstinence period, current smoking, and time of collection of urine sample (*n* = 132).

Aside from a suggestive positive association with FAI, categories of urinary BP were also not associated with hormone levels or semen quality parameters (Table 4). However, BP categories were associated with a dose-related increase in Tail% (*p* for trend = 0.03). Overall, the results for BP were also consistent when modeling geometric mean or SG-corrected paraben concentrations (data not shown). Because we previously reported a positive association between urinary BPA concentration and Tail% in these same men (Meeker et al. 2010b) and because BP and BPA may impart similar biological effects (e.g., estrogenic), we investigated the possibility of an interaction between the two urinary biomarkers on Tail%. When we included both BP categories and BPA quartiles in the multivariable model, both were associated with significant dose- dependent increases in Tail% (Figure 1). However, we found no evidence of interaction in stratified analyses or when including a BP × BPA interaction term in the model (*p* = 0.25; data not shown), although the statistical power of the test for interaction was low because of a fairly small sample size.

## Discussion

As far as we are aware, this is the first human study to explore relationships between biomarkers of paraben exposure and male reproductive health. With the exception of a suggestive inverse association between MP and TSH, and a suggestive positive association between BP and FAI, we found no evidence for a relationship between MP, PP, or BP and altered hormone levels or conventional semen quality parameters. For sperm DNA damage, we observed a suggestive inverse association between PP and TDM, a suggestive positive association between MP and Tail%, and a statistically significant positive association between BP and Tail%. The relationship between BP and Tail% was not confounded by our recently reported relationship between urinary BPA concentrations and Tail% and may suggest additive effects on Tail% in relation to combined exposures to both BP and BPA. We also found an inverse relationship between BMI and urinary paraben concentrations, which may reflect differences in exposure (e.g., product use, diet) and/or paraben metabolism between people with differing BMI.

Our observation that the only statistically significant relationship involved BP is consistent with *in vitro* and animal data that suggest BP has greater reproductive toxicity potential than the other parabens examined in this study (Darbre and Harvey 2008; Golden et al. 2005). However, we did not observe inverse relationships between BP and sperm concentration and testosterone, which were reported in subchronic studies of rodents exposed to BP at around 4 weeks of age (Oishi 2001, 2002a) or *in utero* (Kang et al. 2002). Studies investigating whether BP causes DNA damage are limited, although BP has been associated with genotoxicity in CHO-K1 Chinese hamster ovary cells (Tayama et al. 2008) and increased cell death and injury in rat hepatocytes [National Toxicology Program (NTP) 2004]. Our observation of a positive association with sperm DNA damage, as measured by Tail%, may also be consistent with previous reports that BP may be a suitable vaginal contraceptive because of its ability to inhibit acrosin (an enzyme that aids sperm penetration into the oocyte during the fertilization process), most likely by damaging the sperm membrane (Song et al. 1991, 1989). For example, the primary cause of sperm DNA damage is likely to be oxidative stress (Aitken and De Iuliis 2010; Aitken et al. 2009), which can also damage the sperm membrane (Sharma et al. 2004). Although not well studied, parabens caused oxidative stress in skin cells (Nishizawa et al. 2006), and BP was associated with decreased cellular levels of glutathione and protein-sulfhydryl groups in rat hepatocytes (NTP 2004).

Although it is currently unclear which comet assay parameter is the most relevant measure of sperm DNA damage, Tail% has been shown to be proportional to the frequency of DNA strand breaks (Olive et al. 1990). Tail% may also represent a more sensitive measure of DNA damage than both TDM and comet extent, because Tail% continues to increase with increased DNA damage whereas comet extent may not (McKelvey-Martin et al. 1993). Inconsistent results between the various DNA damage measures obtained by the neutral comet assay regressed on the same independent variable have been observed in previous studies (Meeker et al. 2004b). It has been hypothesized that the different comet assay parameters may reflect different types of DNA strand breaks (Meeker et al. 2004b): Specifically, because of the lack of correlation between TDM and Tail%, a high TDM may be more likely to be associated with double-strand breaks, whereas a high Tail% may reflect single-strand breaks. Thus, in the present study, BP was positively associated with Tail%, which may reflect a relationship between BP and single-strand breaks. However, it is possible that this relationship was a chance finding in our data, and future research is needed in order to confirm our results.

Paraben exposures in the present study were likely representative of those found among men in the U.S. general population. Median and 95th percentile concentrations recently reported in males participating in the National Health and Nutrition Examination Survey (NHANES) for 2005–2006 (Calafat et al. 2010) were, respectively, 23.7 and 491 μg/L for MP, 2.3 and 125 μg/L for PP, and < LOD and 3.2 μg/L for BP, compared with 27.4 and 258 μg/L for MP, 3.5 and 95.5 μg/L for PP, and < LOD and 4.1 μg/L for BP in the present study. It should be noted that paraben exposure was much higher among women than among men in NHANES; females had 75th percentile urinary MP, PP, and BP concentrations that were 4, 7, and 12 times higher, respectively, than those of males (Calafat et al. 2010). Thus, human epidemiologic studies of female reproductive effects and adverse pregnancy outcomes in relation to paraben exposure should also be conducted.

We believe the present study is also the first to assess the temporal variability of paraben concentrations in urine. MP and PP concentrations were weakly correlated in repeated urine samples collected from the same individuals and had low ICCs (≤ 0.35). Repeated BP concentrations were moderately correlated. These measures of temporal reliability were greater than for urinary BPA concentrations among these men (Meeker et al. 2010a) but less than for concentrations of urinary metabolites of phthalates and nonpersistent pesticides among a different subset of men from the ongoing study (Hauser et al. 2004; Meeker et al. 2005). Thus, the lack of an association between urinary parabens and hormone levels or semen quality in the present study may be due to the presence of nondifferential (random) measurement error in our exposure estimates. Future studies should measure paraben concentrations in multiple urine samples collected over the exposure window of interest to reduce exposure measurement error. In the present study, the use of the geometric mean of repeated urinary paraben concentrations, when available, generally resulted in weaker associations with reproductive measures. This was consistent with our recent analysis of urinary BPA among these men (Meeker et al. 2010b). However, this may be because we collected the repeated urine samples weeks or months after the serum/semen samples used for measuring hormone levels, semen quality, and sperm DNA damage, whereas the exposure window of interest would likely be weeks or months leading up to the assessment of these measures.

The present study had a number of other limitations in addition to the likely presence of high temporal variability in paraben exposure levels. This includes the availability of only a single blood or semen sample for the assessment of hormone levels, semen quality, and sperm DNA damage, which may also vary over time. The cross-sectional design of the present analysis also restricts our ability to make conclusions regarding causal relationships, and the relatively small sample size provided low statistical power, which limited our ability to detect subtle relationships between urinary parabens and male reproductive health markers. For example, we observed adjusted regression coefficients of −0.08 and −0.06 for the relationships between sperm concentration and MP or PP, respectively. With our sample size of 190, we would have had 80% power to detect (α = 0.05) adjusted regression coefficients of approximately −0.22 and −0.15, respectively (Lenth 2009). Finally, because of the study’s exploratory nature, we made a large number of statistical comparisons. Thus, we cannot rule out the possibility of chance findings to explain the observed relationship between BP and Tail%.

## Conclusion

We found limited to no evidence of a relationship between paraben concentrations in urine and hormone levels or conventional semen quality parameters. However, it is possible that intraindividual variability in exposure and a modest sample size could have limited our ability to detect subtle relationships. Our observation of a relationship between BP and sperm DNA damage, in addition to the potential for additive effects from combined exposures to BP and BPA, warrants further investigation. Added research on the potential for additive or multiplicative interactions between exposures to multiple environmental agents on male reproduction is also needed.

## Figures and Tables

**Table 1:** Distribution of uncorrected and SG-corrected paraben concentrations in urine (μg/L) collected at the initial clinic visit.

Analyte	*n*	Percent detected	GM	Percentile						Maximum
				10th	25th	50th	75th	90th	95th	
Uncorrected										

MP	194	100	28.6	5.10	11.4	27.4	64.8	191	258	1,080
PP	194	92	3.67	0.40	0.80	3.45	17.1	43.8	95.5	294
BP	194	32	NC	< LOD	< LOD	< LOD	0.30	1.70	4.10	64.5

SG-corrected										

MP	190		35.5	6.63	14.4	32.6	85.7	200	340	1,037
PP	190		4.52	0.48	1.07	4.45	20.9	58.9	107	229
BP	190		NC	< LOD	< LOD	< LOD	0.36	1.60	3.73	32.0

GM, geometric mean. Not calculated (NC) because of the high percentage of samples < LOD. For MP, LOD was 1.0 μg/L; for both PP and BP, LOD was 0.2 μg/L.

**Table 2:** Adjusted^a^ regression coefficients for change in serum hormone level associated with a ln-unit increase in urinary concentrations of MP or PP (*n* = 167).

	MP		PP	
Hormone	β-Coefficient (95% CI)	*p*-Value	β-Coefficient (95% CI)	*p*-Value
FSH (IU/L)^b^	0.01 (−0.06 to 0.09)	0.69	0.01 (−0.04 to 0.06)	0.71
LH (IU/L)^b^	0.02 (−0.04 to 0.09)	0.48	0.01 (−0.03 to 0.05)	0.69
Inhibin B (pg/mL)	2.62 (−8.07 to 13.3)	0.63	−1.15 (−8.35 to 6.06)	0.75
FSH:inhibin B ratio^b^	0.02 (−0.12 to 0.17)	0.74	0.03 (−0.07 to 0.12)	0.61
T (ng/dL)^c^	9.23 (−2.98 to 21.5)	0.14	6.82 (−1.36 to 15.0)	0.10
E_2_ (pg/mL)	−0.12 (−1.74 to 1.51)	0.89	−0.01 (−1.10 to 1.08)	0.98
SHBG (nmol/mL)^b^	0.004 (−0.04 to 0.05)	0.87	0.001 (−0.03 to 0.03)	0.94
FAI^b^	0.02 (−0.02 to 0.07)	0.31	0.02 (−0.01 to 0.05)	0.14
Free T (ng/dL)	0.21 (−0.08 to 0.52)	0.15	0.13 (−0.07 to 0.33)	0.20
T:LH ratio^b^	0.004 (−0.07 to 0.08)	0.92	0.01 (−0.04 to 0.06)	0.56
E_2_:T ratio^b^	−0.05 (−0.12 to 0.03)	0.20	−0.03 (−0.08 to 0.02)	0.31
Prolactin (ng/mL)^b^	−0.01 (−0.07 to 0.04)	0.60	0.01 (−0.03 to 0.04)	0.65
Free T_4_ (ng/dL)	−0.01 (−0.03 to 0.02)	0.67	0.01 (−0.01 to 0.03)	0.47
Total T_3_ (ng/mL)	0.005 (−0.02 to 0.02)	0.63	0.003 (−0.01 to 0.02)	0.64
TSH (μIU/mL)^b^	−0.07 (−0.15 to 0.01)	0.08	−0.02 (−0.07 to 0.03)	0.46

Abbreviations: CI, confidence interval, T, testosterone.

a Adjusted for SG, age, BMI, current smoking status, and time of day of blood/urine sample collection.

b Model included ln-transformations for parabens and hormones; inhibin B, testosterone, free T, E_2_, free T_4_, and total T_3_ were modeled untransformed.

c Models for T also adjusted for ln-transformed SHBG.

**Table 3:** Adjusted^a^ linear regression coefficients for change in semen quality or sperm measures associated with a ln-unit increase in urinary concentrations of MP or PP (*n* = 190).

	MP		PP	
Semen/sperm measure	β-Coefficient (95% CI)	*p*-Value	β-Coefficient (95% CI)	*p*-Value
Semen quality				

Total count (10^6^)^b^	−0.07 (−0.25 to 0.11)	0.42	−0.05 (−0.17 to 0.08)	0.45
Concentration (10^6^/mL)^b^	−0.08 (−0.24 to 0.08)	0.33	−0.06 (−0.17 to 0.05)	0.27
Motility (% motile)	−0.48 (−3.26 to 2.30)	0.73	−0.20 (−2.09 to 1.69)	0.84
Morphology (% normal)	0.04 (−0.52 to 0.60)	0.89	0.14 (−0.24 to 0.52)	0.46

Sperm motion				

VSL (μm/sec)	0.001 (−1.72 to 1.73)	1.00	−0.05 (−1.22 to 1.12)	0.93
VCL (μm/sec)	−0.17 (−3.09 to 2.75)	0.91	−0.18 (−2.17 to 1.80)	0.86
Linearity (%)	−0.25 (−2.00 to 1.50)	0.78	0.002 (−1.19 to 1.19)	1.00

DNA damage^c^				

Comet extent (μm)	0.67 (−6.10 to 7.45)	0.84	−1.19 (−5.61 to 3.22)	0.59
TDM (μm)	−1.31 (−3.58 to 0.95)	0.25	−1.42 (−2.88 to 0.04)	0.06
Tail%	2.35 (−0.16 to 4.85)	0.07	0.67 (−0.98 to 2.32)	0.42

CI, confidence interval.

a Adjusted for SG, age, BMI, abstinence period, current smoking, and time of day of urine/semen sample collection.

b Model included ln-transformations of parabens, sperm count, and sperm concentration.

c *n* = 132.

**Table 4:** Adjusted linear regression coefficients for change in hormone levels (*n* = 167), semen quality (*n* = 190), and sperm DNA damage measure (*n* = 137) associated with categories of urinary BP.

Measure	β-Coefficient (95% CI)
Serum hormone levels^a^	

FSH (IU/L)^b^	0

< 0.2 μg/L	−0.04 (−0.29, 0.21)
0.2–0.6 μg/L	−0.05 (−0.31, 0.21)
> 0.6 μg/L	0.65
*p* for trend	

LH (IU/L)^b^	

< 0.2 μg/L	0
0.2–0.6 μg/L	−0.12 (−0.33, 0.08)
> 0.6 μg/L	0.04 (−0.18, 0.26)
*p* for trend	0.99

Inhibin B (pg/mL)	

< 0.2 μg/L	0
0.2–0.6 μg/L	0.81 (−35.3, 36.9)
> 0.6 μg/L	28.4 (−9.30, 66.0)
*p* for trend	0.18

FSH:inhibin B ratio^b^	

< 0.2 μg/L	0
0.2–0.6 μg/L	0.07 (−0.42, 0.55)
> 0.6 μg/L	−0.15 (−0.65, 0.36)
*p* for trend	0.66

T (ng/dL)	

< 0.2 μg/L	0
0.2–0.6 μg/L	−13.7 (−54.4, 27.0)
> 0.6 μg/L	37.6 (−4.74, 79.9)
*p* for trend	0.17

E_2_ (pg/mL)	

< 0.2 μg/L	0
0.2–0.6 μg/L	−0.70 (−6.05, 4.65)
> 0.6 μg/L	−2.95 (−8.54, 2.63)
*p* for trend	0.32

SHBG (nmol/mL)^b^	

< 0.2 μg/L	0
0.2–0.6 μg/L	−0.11 (−0.26, 0.04)
> 0.6 μg/L	−0.07 (−0.23, 0.09)
*p* for trend	0.24

FAI^b^	

< 0.2 μg/L	0
0.2–0.6 μg/L	0.05 (−0.10, 0.19)
> 0.6 μg/L	0.15 (−0.003, 0.31)
*p* for trend	0.06

Free T (ng/dL)	

< 0.2 μg/L	0
0.2–0.6 μg/L	−0.28 (−1.26, 0.71)
> 0.6 μg/L	0.72 (−0.31, 1.75)
*p* for trend	0.29

T:LH ratio^b^	

< 0.2 μg/L	0
0.2–0.6 μg/L	0.05 (−0.19, 0.30)
> 0.6 μg/L	0.04 (−0.22, 0.30)
*p* for trend	0.69

E_2_:T ratio^b^	

< 0.2 μg/L	0
0.2–0.6 μg/L	0.06 (−0.19, 0.30)
> 0.6 μg/L	−0.23 (−0.48, 0.03)
*p* for trend	0.15

Prolactin (ng/mL)^b^	

< 0.2 μg/L	0
0.2–0.6 μg/L	−0.004 (−0.18, 0.18)
> 0.6 μg/L	−0.05 (−0.24, 0.14)
*p* for trend	0.62

Free T_4_ (ng/dL)	

< 0.2 μg/L	0
0.2–0.6 μg/L	−0.04 (−0.14, 0.05)
> 0.6 μg/L	−0.003 (−0.10, 0.10)
*p* for trend	0.76

Total T_3_ (ng/mL)	

< 0.2 μg/L	0
0.2–0.6 μg/L	−0.01 (−0.07, 0.06)
> 0.6 μg/L	0.01 (−0.06, 0.08)
*p* for trend	0.86

TSH (μIU/mL)^b^	

< 0.2 μg/L	0
0.2–0.6 μg/L	−0.004 (−0.26, 0.26)
> 0.6 μg/L	−0.12 (−0.39, 0.15)
*p* for trend	0.42

Semen quality and sperm DNA damage^c^	

Total count (10^6^)^b^	

< 0.2 μg/L	0
0.2–0.6 μg/L	−0.23 (−0.90, 0.43)
> 0.6 μg/L	0.18 (−0.49, 0.84)
*p* for trend	0.80

Concentration (10^6^/mL)^b^	

< 0.2 μg/L	0
0.2–0.6 μg/L	−0.28 (−0.90, 0.33)
> 0.6 μg/L	0.20 (−0.34, 0.73)
*p* for trend	0.65

Motility (% motile)	

< 0.2 μg/L	0
0.2–0.6 μg/L	−1.33 (−12.0, 9.29)
> 0.6 μg/L	6.25 (−3.04, 15.5)
*p* for trend	0.24

Morphology (% normal)	

< 0.2 μg/L	0
0.2–0.6 μg/L	0.60 (−1.56, 2.76)
> 0.6 μg/L	0.40 (−1.49, 2.28)
*p* for trend	0.61

VSL (μm/sec)	

< 0.2 μg/L	0
0.2–0.6 μg/L	−2.40 (−8.98, 4.17)
> 0.6 μg/L	3.99 (−1.75, 9.73)
*p* for trend	0.28

VCL (μm/sec)	

< 0.2 μg/L	0
0.2–0.6 μg/L	−5.57 (−16.7, 5.55)
> 0.6 μg/L	6.11 (−3.61, 15.8)
*p* for trend	0.37

Linearity (%)	

< 0.2 μg/L	0
0.2–0.6 μg/L	−6.71 (−13.4, −0.07)
> 0.6 μg/L	−0.25 (−6.05, 5.55)
*p* for trend	0.58

Comet extent (μm)	

< 0.2 μg/L	0
0.2–0.6 μg/L	1.38 (−22.1, 24.8)
> 0.6 μg/L	3.09 (−20.5, 26.6)
*p* for trend	0.79

TDM (μm)	

< 0.2 μg/L	0
0.2–0.6 μg/L	−1.10 (−8.97, 6.78)
> 0.6 μg/L	−2.91 (−10.8, 5.00)
*p* for trend	0.46

Tail%	

< 0.2 μg/L	0
0.2–0.6 μg/L	6.81 (−1.80, 15.4)
> 0.6 μg/L	8.23 (−0.41, 16.9)
*p* for trend	0.03

Abbreviations: CI, confidence interval; T, testosterone.

a Adjusted for SG, age, BMI, current smoking, and time of day of urine/serum/semen sample collection.

b Adjusted for SG, age, BMI, abstinence period, current smoking, and time of urine sample.

c Model included ln-transformations.
